# Effect of nano-TiO_2_ size and utilization ratio on the performance of photocatalytic concretes; self-cleaning, fresh, and hardened state properties

**DOI:** 10.1007/s11356-024-33660-9

**Published:** 2024-05-18

**Authors:** Hatice Gizem Şahin, Müge Temel, Gözde Koçak, Ali Mardani, Ali Kara

**Affiliations:** 1 Department of Civil Engineering, Bursa Uludag University, Bursa, Turkey; 2 Department of Chemistry, Bursa Uludag University, Bursa, Turkey

**Keywords:** Photocatalyst, Reactive Black 5, Nano-TiO_2_, Dye removal, Compressive-flexural strength, Bohme abrasion resistance

## Abstract

In this study, photocatalysis technology was used to reduce water pollution. Decolorization of Reactive Black 5 using nano-TiO_2_ (NT) as a photocatalyst was investigated by adsorption and degradation experiments. Effects of NT particle size and utilization ratio on the time-dependent flow performance, compressive-flexural strength, and Bohme abrasion resistance of cementitious systems were investigated. In addition to the NT-free control mixture, a total of six photocatalytic self-cleaning mortar mixtures (PSCM) were prepared using NT in two different particle sizes (28 and 38 nm) and three different ratios (0.5%, 1%, and 1.5%). The PSCM sample containing 38 nm NT exhibited superior performance in terms of photocatalytic properties compared to the 28 nm state. It was observed that the flow performance of PSCM mixtures with NT substitution is adversely affected regardless of the NT type. Mixtures containing NT with a lower particle size (28 nm) had higher compressive and flexural strengths.

## Introduction

Air and water pollution caused by rapidly developing industrialization brings along important social concerns (Kalıpcılar et al. 2016; Mardani-Aghabaglou et al. 2019; Sezer et al. 2016; Yiğit et al. 2020; Mardani-Aghabaglou 2016; Yüksel et al. 2016; Şahin and Mardani 2022a). At the forefront of these concerns is “health problems.” It is known that approximately 9% of the total annual carbon emissions come from the construction sector. Studies are being carried out on CO_2_ emissions resulting from increasing environmental requirements and different suggestions have been presented to reduce CO_2_ emissions. In a very comprehensive study conducted by Riekstins et al. (2020), it was stated that one of these is to ensure energy efficiency by using grinding aids during clinker grinding, and another is to reduce cement usage by substituting certain amounts of mineral additives into the cement used. The use of mineral admixtures in cementitious systems is widely researched (Şahin et al. 2024a, 2024b).

Volatile organic compounds and inorganic oxides (CO_2_, NO_*x*_, and SO_*x*_) in the air accelerate global warming by causing secondary hazards such as acid rain in addition to health problems (Nath et al. 2016). The application of “photocatalysis” technology, which accelerates the natural decomposition process, was accepted as an effective solution to reduce and/or prevent the pollution in question (Yang et al. 2000). With the help of this technology, numerous pollutants, including hydrocarbons, chlorinated hydrocarbons, SO_2_, CO, and NO, can be transformed directly into H_2_O and CO_2_ without the use of an additional carrier gas (Castro-Hoyos et al. 2022). In a study by Beeldens (2008), it was stated that concrete is an ideal substrate for “photocatalysis” reactions due to its large surface area. Similarly, it was stated by various researchers that the self-cleaning and pollutant removal performance is improved when photocatalytic materials are used in concrete (Liang et al. 2019). In this process, energy and time savings are achieved due to the use of sun rays and rain water (Obuchi et al. 1999; Yu and Brouwers 2009). Due to these positive effects, concretes with self-cleaning technology, produced by using materials with photocatalytic properties, have become a popular topic in recent years (Shen et al. 2015; Zailan et al. 2017). Most studies in this area have focused on the use of semiconductor oxides as photocatalysts, such as titanium dioxide (TiO_2_), zinc oxide (ZnO), cadmium selenide (CdSe), and tungsten oxide (WO_3_). Compared to other oxides, TiO2 is preferred more because of (i) low cost, (ii) non-toxicity and (iii) good thermal stability, (iv) easy accessibility, and (v) chemical-biological inertness (Yuranova et al. 2007; Yasmina et al. 2014; Lazar et al. 2012; Kweinor Tetteh et al. 2021; Zhang et al. 2016). Apart from its self-cleaning effect, it was reported that the use of nano-TiO_2_ (NT) has some positive effects on the mechanical properties of cementitious systems (Sanchez and Sobolev 2010; Pacheco-Torgal and Jalali 2011). In a study conducted by Li et al. (2007), it was determined that the flexural-fatigue performance of the concrete mixture increased with the addition of 1% NT. In a study by Daniyal et al. (2019), it was emphasized that the use of NT improves the microstructure by causing a denser matrix formation. In another study by Senff et al. (2012), it was determined that the use of NT increased the compressive strength of concrete mixtures.

The large amount of waste water resulting from industrialization and globalization causes water pollution by being discharged into water resources without undergoing treatment processes. It was emphasized that dyestuffs come first among these pollutants (Lellis et al. 2019). It was declared that the mentioned dyestuffs are classified as nitro, azo, indigo, phtalein, anthraquinone, triphenyl, methyl, and nitrate dyes according to the chemical structures of the chromophore groups. Reactive Black 5 (RB5) dyestuff is in the azo dyestuffs class, which constitutes approximately 70% of the dyestuffs used in the industry (Berradi et al. 2019). It was emphasized that RB5 is a water-soluble synthetic dyestuff found most commonly in wastewater (Jalali Sarvestani and Doroudi 2020). It was understood that the chemical structure of RB5 is characterized by an azo (-N = N-) chromophore group and a sulfonic (-SO3-) functional group (Przystas et al. 2012; Sudha et al. 2014; Benkhaya et al. 2017; Kaplan et al. 2019). Dyestuffs cause deterioration of water quality, decrease in gas solubility, increase in toxicity, allergic reactions, and cancer in the skin by reducing photosynthesis in the aquatic ecosystem (Sudha et al. 2014; Asad et al. 2007; Shanehsaz et al. 2015; Imran et al. 2015; Slama et al. 2021). For this reason, the treatment of dyestuffs from wastewater has become extremely important.

Traditional treatment methods are used to reduce the environmental impact of dyestuffs. It was reported that these methods are (i) ozonation (Snider and Porter 1974), (ii) chlorination (Francy et al. 2012), (iii) sedimentation (Mazari and Abdessemed 2020), (iv) ultrafiltration (Barredo-Damas et al. 2010), and (v) adsorption (Ram et al. 2012; Georgiou et al. 2002).

It was emphasized that traditional treatment methods are not sufficient to remove dyestuffs, despite the various positive effects they provide. Advanced oxidation processes have come about as a result of this. It was understood that photocatalytic decolorization is an advanced oxidation process and is a more effective and sustainable method for the removal of dyestuffs compared to other methods (Natarajan et al. 2018). As emphasized earlier, this process converts organic pollutants into non-toxic small molecules such as CO_2_, H_2_O, and HCl using low-energy UV light and a semiconductor (Espulgas et al. 2002; Bizani et al. 2006; Cebeci and Selçuk 2020).

In this study, photocatalysis technology was used to reduce water pollution. It was reported that charcoal (Horgnies et al. 2012), methylene blue (Zhou et al. 2022), and Rhodamine-B (Ruot et al. 2009) are used in concrete structures to control this process. However, in the textile industry, RB5 was found to be removed from fabrics by photocatalysis technology (Tang and Chen 2004). It was emphasized before that RB5 is found in high amounts in wastewater as a dyestuff. In this study, it was planned to remove RB5 by applying the photocatalysis process. Thus, it is foreseen that the quality of wastewater will be increased by applying the photocatalysis process. However, the use of NT is also aimed to improve the mechanical properties of the produced photocatalytic self-cleaning mortar (PSCM) mixtures. Within the scope of this study, it was aimed to examine the effect of NT particle size and utilization ratio on photocatalytic concrete properties. In addition to the control mixture without NT, six series of NT substituted mixtures were prepared. For this purpose, PSCM mixtures were produced by replacing NT with particle sizes of 28 nm and 38 nm with cement at the rates of 0, 0.5, 1, and 1.5%. Time-dependent flow performance, photocatalytic property, compressive-flexural strength, and Böhme abrasion resistance of PSCM mixtures were determined. The photocatalytic property of the mixtures was examined in two stages: adsorption and decolorization.

## Material and method

### Materials

CEM I 42.5R type PC was used as a binder. The properties of the cement are shown in Table 1.

**Table 1:** Some properties of cement

Oxides (%)		Cement
SiO_2_		18
Al_2_O_3_		4.75
Fe_2_O_3_		3.58
CaO		63
MgO		1.4
Na_2_O + 0.658 K_2_O		0.7
SO_3_		3.11
Specific gravity		3.06
Specific surface area (cm^2^/g)		3441
Compressive strength (MPa)	7 days	42.8
	28 days	51.8
Setting time (min)	Initial	170
	Final	240

Two NT with 28- and 38-nm particle size were used in order to fully comprehend the impact of NT fineness on the performance of cementitious systems. Some properties of the NT used are shown in Table 2.

**Table 2:** Some properties of NT used in the study

Value	Units	28 nm NT	38 nm NT
Purity	%	> 99	> 99
Size	nm	28	38
Specific surface area	m^2^/g	> 60	35
Loss of weight in drying	%	2 max	1.2
Loss of weight in ignition	%	5 max	3.2
pH	-	5.5–7.0	5.5–6.5
Color	-	White	White

**Fig. 1:**
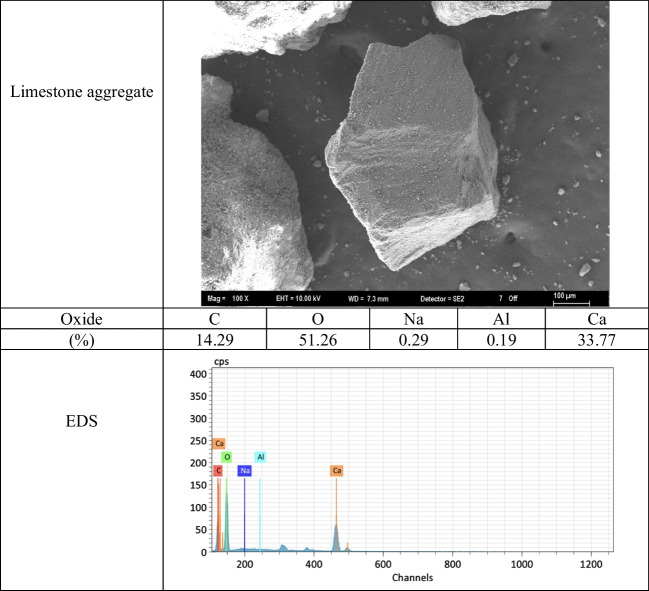
SEM and EDS analysis of limestone aggregate

**Fig. 2:**
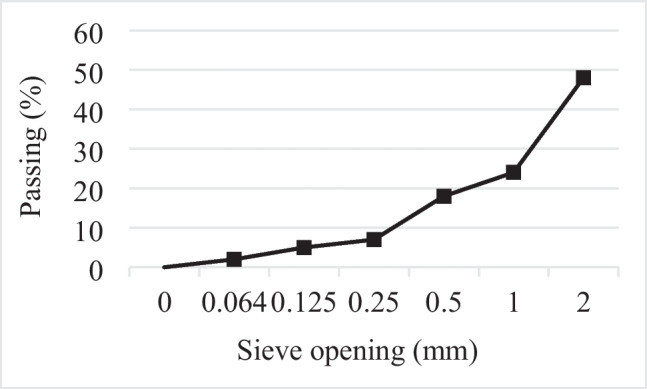
Grading curve of the aggregate used in the study

Crushed limestone aggregate with a *D*_max_ of 2 mm was used in the preparation of photocatalytic self-cleaning mortar (PSCM) mixtures. The specific gravity and water absorption capacity values of the aggregate determined in accordance with TS EN 1097–6 were measured as 2.58 and 0.4%, respectively. Figure 1 shows the SEM analysis image and EDS results of the limestone aggregate. Additionally, the granulometry of the aggregate is shown in Fig. 2.

A polycarboxylate ether-based high range water reducing admixture (HRWRA) was used to achieve a target flow value of 240 ± 20 mm. Some properties of the HRWRA are shown in Table 3.

**Table 3:** Some properties of HRWRA

Density (g/cm^3^)	Solid content (%)	pH	Chlorine content (%)	Na_2_O ratio (%)
1.060	32	2–5	< 0.1	< 10

**Fig. 3:**
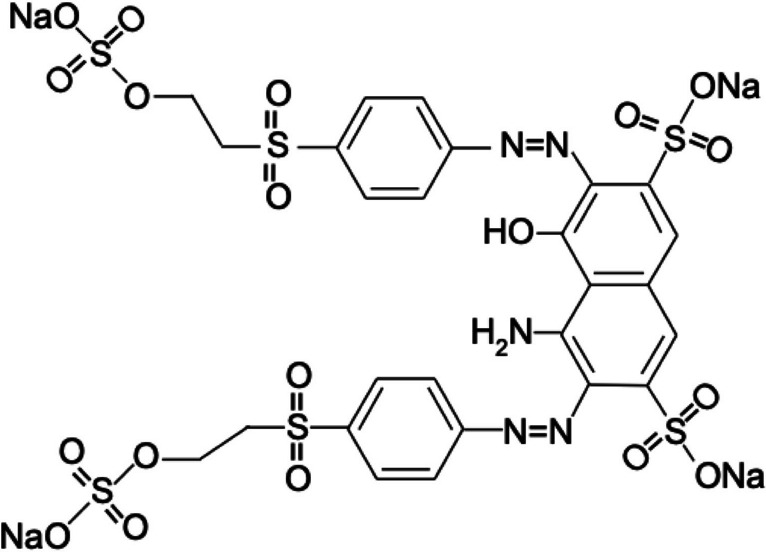
Reactive Black 5

In experimental studies, RB5 (dye content ≥ 50%) used to determine photocatalytic properties, from Sigma-Aldrich, polyvinyl alcohol (PVA) (Mw = 70.000, ≥ 85% hydrolyzed), toluene (≥ 99%), HCl (32%), benzoyl peroxide (with 25% H_2_O), NaOH (98%, pellet), and ethyl alcohol were obtained from Merck. The chemical structure of the RB5 dyestuff applied in the study is shown in Fig. 3.

### Mixing ratios

In order to examine the effect of NT particle size and utilization ratio on the photocatalytic concrete properties, six series of NT substituted mixtures were prepared in addition to the NT-free control mixture. For this purpose, PSCM mixtures were produced by replacing NT with 28-nm and 38-nm particle sizes with 0, 0.5, 1, and 1.5% cement. Within the scope of the study, the amount of material used in the production of 1 m^3^ PSCM mixtures with a flow value of 240 ± 20 mm is shown in Table 4. The w/b ratio was kept as 0.45 in all mixtures. The naming of the mixtures was made according to the NT particle size and utilization ratio. For example, the mixture containing NT at a 1% substitution ratio with a particle size of 28 nm was named NT28_1%. The prepared samples were subjected to water curing in accordance with the standard until the test day.

**Table 4:** Amount of material used in the production of 1 m^3^ PSCM (kg/m^3^)

Mixture	Cement	Nano-TiO_2_ (%)*	Aggregate	HRWRA	w/b
Control	550	0	1358.43	3.5	0.45
NT28_0.5%	547.75	0.5	1357.43	4	
NT28_1%	544.50	1	1357.69	4.3	
NT28_1.5%	541.75	1.5	1357.79	4.5	
NT38_0.5%	547.75	0.5	1358.58	3.5	
NT38_1%	544.50	1	1358.85	3.8	
NT38_1.5%	541.75	1.5	1358.95	4.0	

^*^By cement weight

### Method

**Fig. 4:**
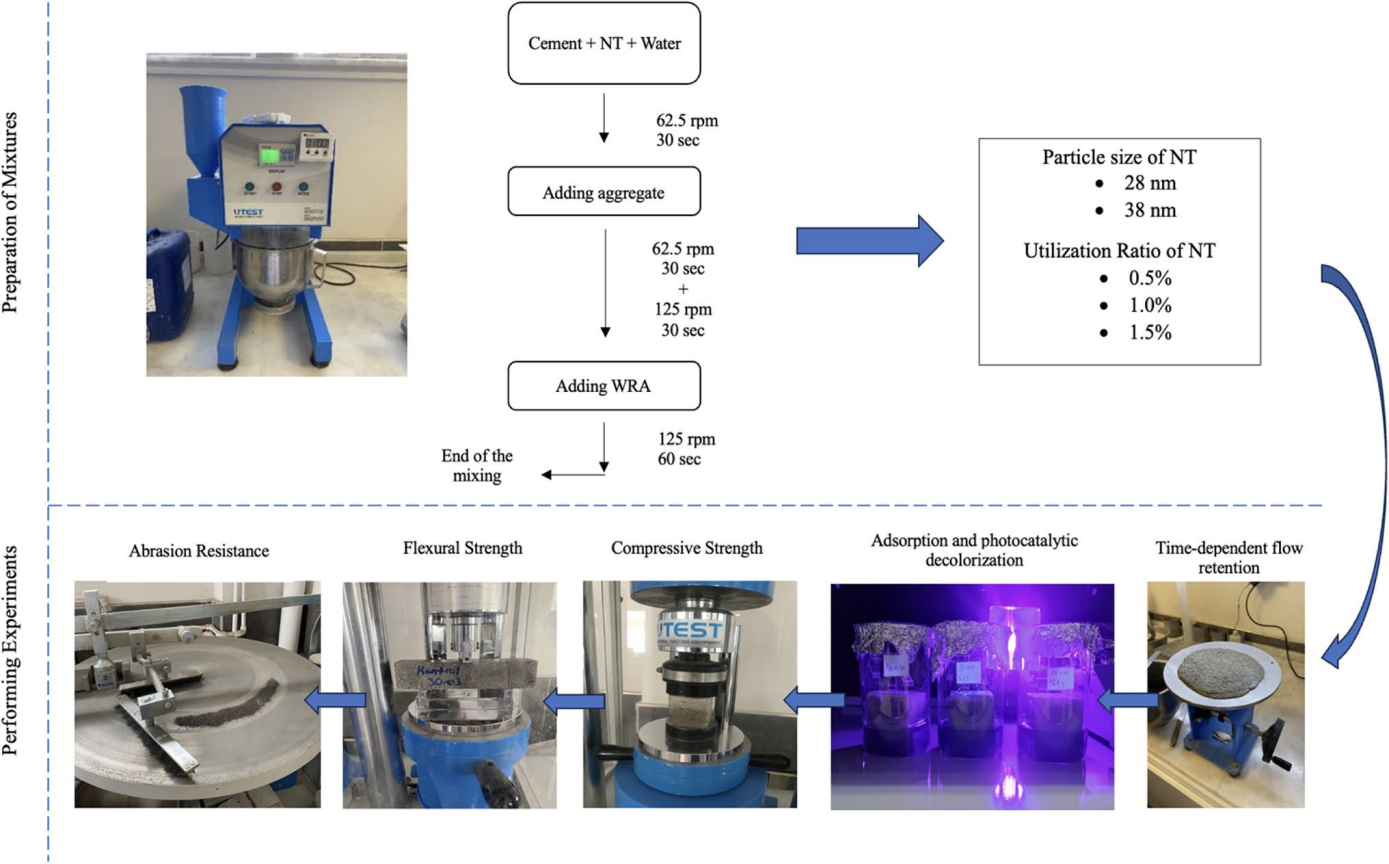
Workflow carried out within the scope of the study

Time-dependent flow performance, photocatalytic property, compressive-flexural strength, and Bohme abrasion resistance of PSCM mixtures were determined. The workflow applied within the scope of the study is shown in Fig. 4.

#### Flow performance

Nano-materials, which are highly reactive and increase the risk of agglomeration (Wiesner and Bottero 2017), can seriously affect fresh state properties such as time-dependent flow and rheological performance of cementitious systems (Nazar et al. 2020). Thus, it was thought that the properties that provide information about the homogeneity and flowability of cementitious systems should be determined in mixtures containing NT (Jiang et al. 2018; Senff et al. 2012). It was understood that these properties of paste mixtures are generally determined by rheology testing (Şahin and Mardani 2022b, Şahin and Mardani 2023a). It was understood that these properties of mortar and concrete mixtures are generally determined by time-dependent flow performance. According to the literature, it was reported that the rheological properties are generally negatively affected by the addition of nano-materials to the mixture. However, some studies have also been found that state the opposite of this situation. In order to resolve these contradictions, time-dependent flow properties of mortar mixtures were investigated.

Time-dependent flow performance of PSCM mixtures was determined by measuring the flow value in accordance with ASTM C1437. In addition, the flow value was measured time interval 20 min for 60 min in order to examine the effect of NT usage on the flow performance of the mixtures.

#### Photocatalytic properties

Photocatalytic properties of the mixtures were investigated in two stages as adsorption and decolorization.

##### Adsorption experiments

**Fig. 5:**
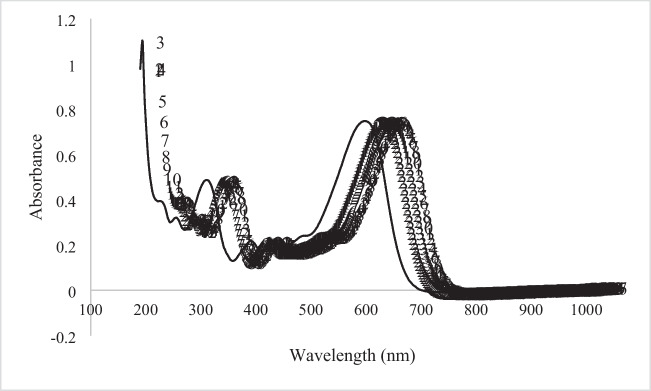
Wavelength-absorbance graph of RB5

It was reported by Ferkous et al. (2022) that the pH value of the solution is the most important factor affecting the adsorption and decolorization of dyestuffs on concrete samples, since the surface charge of the adsorbent changes. For the adsorption and decolorization studies of RB5 dyestuff, the optimum pH value was chosen as pH3 based on previous studies. Three different specimens, control, NT28_1.5%, and NT38_1.5%, were used for adsorption studies of concrete samples. Experimental studies were carried out under the optimum conditions of 25 °C ambient temperature and 30 mg/L solution concentration; it was carried out by taking 50 ml of dyestuff solution. The surface area of the concrete specimens in contact with the dye solution was measured as 75 cm^2^. RB5 dyestuff solutions containing concrete samples were kept in the dark for 24 h. At the end of the experiment, the remaining dyestuff concentration in the solutions was determined by UV–vis spectrophotometer (Shimadzu-2100 UV–vis, Japan). In Fig. 5, the wavelength-absorbance graph of the RB5 dyestuff is shown. The adsorption capacity (*Q*_*e*_) (mg/g) of the remaining dyestuff concentration in each solution was determined using Eq. 1 (Özer et al. 2015; El-Bery et al. 2022).

1 $${Q}_{e}=\frac{\left({C}_{0}-{C}_{e}\right)v}{m}$$

where ${C}_{0}$ is the initial dye concentration (mg/L), ${C}_{e}$ is the dye remaining concentration in solution (mg/L), $v$ is the volume of solution (mL), and $m$ is the polymer amount (g).

##### Decolorization experiments

Photocatalytic decolorization of RB5 dyestuff was investigated over time. The experiments were carried out under UV light with a wavelength of 366 nm and in a cabinet at a constant temperature of 25 °C. The remaining dyestuff concentration in the solution was determined by UV–vis spectrophotometer. The % removal amount was calculated using Eq. 2 (Elhadj et al. 2020).

2 $$\mathrm{\%}{\mathrm{Removal}}=\frac{\left({C}_{0}-{C}_{e}\right)x100}{{C}_{e}}$$

where ${C}_{0}$ is the initial dye concentration (mg/cm^2^), and ${C}_{e}$ is the dye remaining concentration in solution (mg/L).

**Fig. 6:**
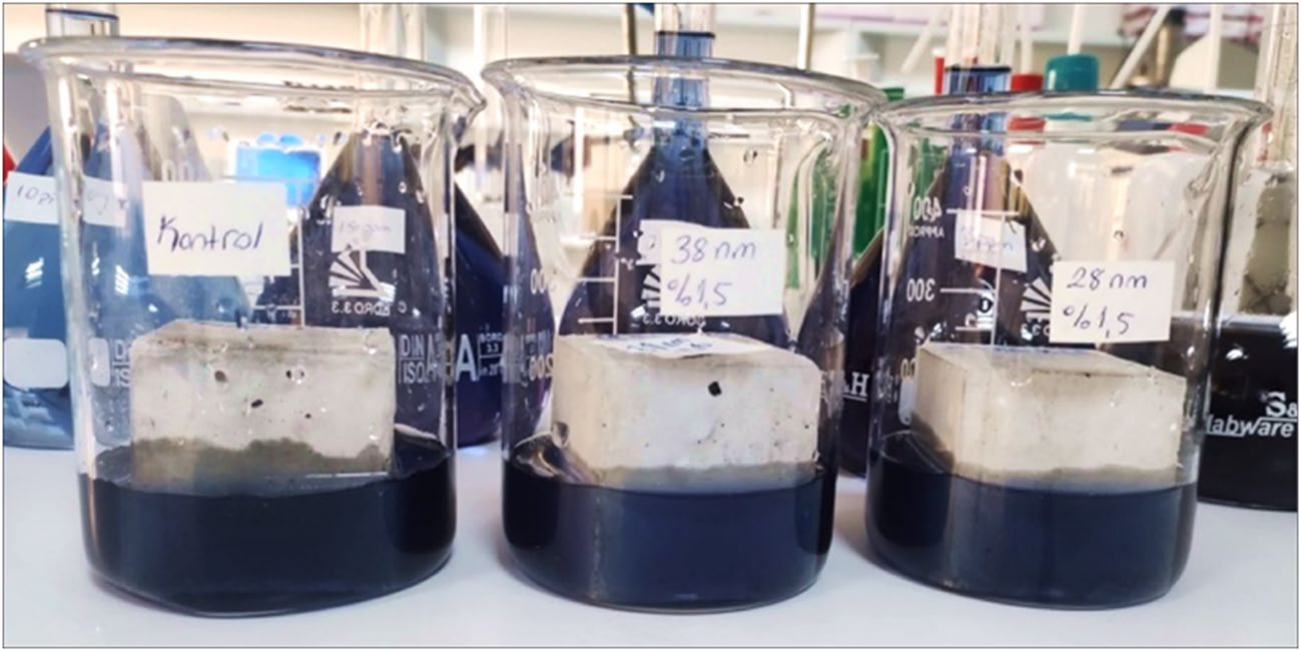
Adsorption of RB5 dyestuff to PSCM samples

The adsorption of RB5 dyestuff to PSCM samples is shown in Fig. 6.

**Fig. 7:**
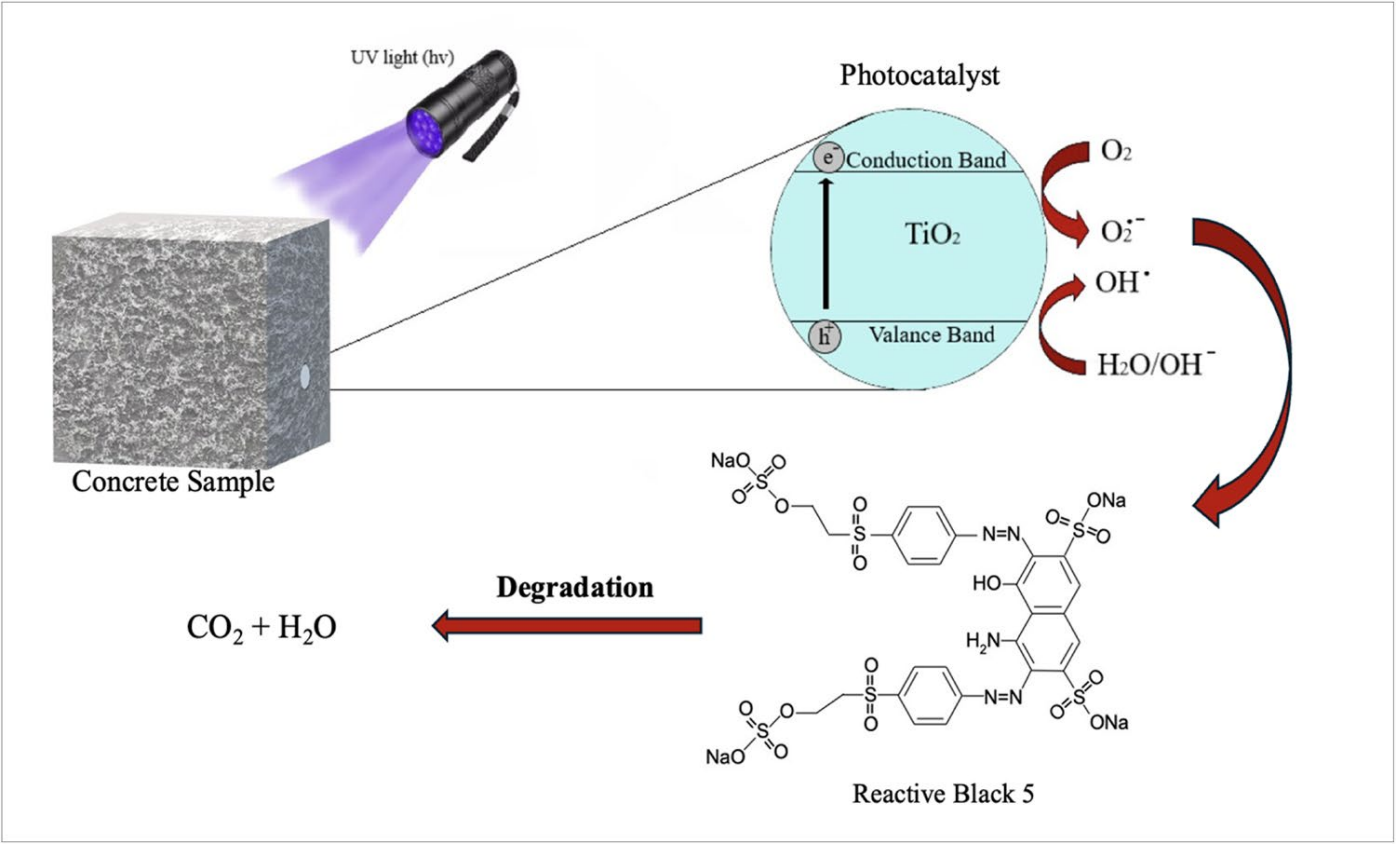
Schematic representation of the photocatalytic degradation mechanism of RB5 dyestuff with concrete sample

A schematic depiction of the photocatalytic degradation mechanism of RB5 dyestuff with a concrete sample is presented in Fig. 7. Photocatalytic reactions commence with the excitation of the TiO_2_ semiconductor by a UV source. The electrons in the valence band move to the conduction band, creating positive vacancies (h +) in the valence band and electrons (e^−^) in the conduction band. These electron–hole pairs initiate redox reactions by transferring to the surface of the TiO_2_ photocatalyst. Thus, water molecules or hydroxyl (OH^−^) ions in the valence band are oxidized by the vacancies to form active hydroxyl radicals (OH). The electrons transitioning to the conduction band also react with O_2_ molecules on the photocatalyst surface to produce O_2_^−^. The free radicals facilitate the degradation of pollutant compounds, leading to their conversion into CO_2_ and H_2_O (Koçak Mutlu et al. 2024; Navidpour et al. 2023). This mechanism generally describes not only the removal of pollution in water (dyes, lead, copper, mercury, etc.) but also the elimination of volatile organic compounds in air (benzene, formaldehyde, toluene, etc.) and the self-cleaning ability of the TiO_2_ semiconductor with the assistance of UV rays (Tsang et al. 2019).

#### Mechanical properties

The 7- and 28-day compressive strength and Bohme abrasion resistance of the mixtures were determined on 50-mm cube samples, respectively, according to ASTM C109 and EN 1338 Standards. The 7- and 28-day flexural strength of the mixtures was determined by performing a three-point bending test on 40 × 40 × 160 mm prism specimens in accordance with TS EN 196–1 Standard.

## Experimental results and discussion

### Photocatalytic properties

**Fig. 8:**
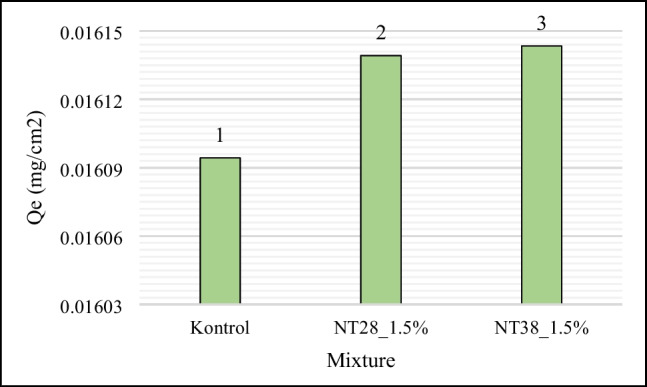
Adsorption capacity

**Fig. 9:**
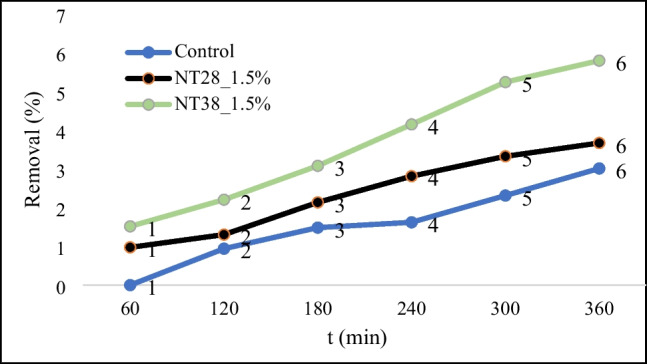
Graph of % removal over time

RB5 adsorption capacities and photocatalytic decolorization of control, NT28_1.5%, and NT38_1.5% are shown in Figs. 8 and 9, respectively.

Regardless of the particle size, the adsorption capacity of the mixtures containing NT was found to be higher than the control mixture. Also, it is seen that the 38 nm NT-containing PSCM sample has better RB5 adsorption capacity and dye removal performance than the 28-nm case. It was stated by Gunnelius et al. (2014) that the surface area and activity of NT increase with increasing particle fineness. It is thought that the hydration rate is higher in mixtures containing NT with a particle size of 28 nm (Li et al. 2020). It was also stated by Nazari and Riahi (2010) and Nazari et al. (2010) that with the decrease in the particle fineness of NT, the potential of NTs that do not or only slightly bind to CH to leak to the surface is high. In this case, it is thought that the particles infiltrating the surface increase the photocatalytic effect. As expected, the percentage of dye removal increased over time.

In a study by Kalkan et al. (2014), the removal of RB5 from aqueous solutions using silica fume after its modification with laccase from Russulaceae (*Lactarius volemus*) was examined. It was shown that the adsorption experimental data agreed well with the Langmuir isotherm model, and the adsorption capacity was found to be 322.58 mg/g. As a result, it has been understood that laccase-modified silica fume can be used as an alternative low-cost adsorbent in the processing of aqueous solutions. In another study conducted by Erdal et al. (2010), the removal of textile dye RB5 color by actively growing the mycelium of the *Penicillium chrysogenum* MT-6 fungus isolated from cement-contaminated soil was examined. The maximum removal/uptake of dye by the fungus was measured to be 89% with a biomass production of 3.83 g/l at an initial dye concentration of 0.3 g/l in 100 h. As a result, it was determined that the fungus is a good biosystem for decolorizing the medium containing RB5.

In order to explain the decolorization kinetics of RB5 dyestuff in the presence of concrete samples, a suitable kinetic model was searched by drawing ln(*C*/*C*_*0*_) − *t* graphs. The degradation kinetics of RB5 dyestuff were also evaluated by literature studies and explained with a first-order kinetic model (Hang et al. 2005).

3 $$r=\frac{-dC}{dt}={K}_{{\mathrm{app}}}.C$$

where *r* is the rate and *K*_app_ (min^−1^) the apparent first-order rate constant; the integration of Eq. (4) gives:

4 $${\mathrm{ln}}\frac{C}{{C}_{0}}=-{K}_{{\mathrm{app}}}.t$$

**Fig. 10:**
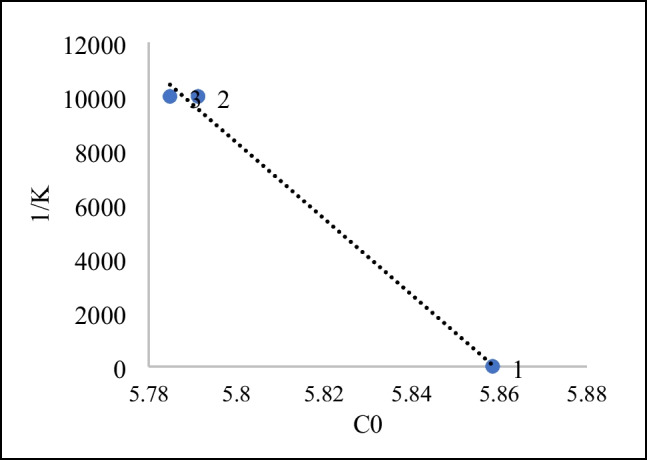
1/*K*_app_-C graph

It is known that the ln(*C*/*C*_*0*_) graph is linear and examined as a function of time (David and Vedhi 2017). A constant *K*_app_ value is characteristic of the photocatalytic process and defines that the model conforms to the Langmuir–Hinshelwood model, where the reaction takes place in diluted medium (Elhadj et al. 2020). The graph of 1/*K*_app_ vs. *C*_0_ is presented in Fig. 10.

#### Modeling of degradation kinetics by the Langmuir–Hinshelwood

The Langmuir–Hinshelwood (L–H) model states that dyestuffs are adsorbed according to the Langmuir model before undergoing photocatalytic degradation (Elhadj et al. 2020). Adsorption of RB5 dyestuff on the surface of concrete samples affects the rate of degradation. The L–H equation, which expresses the connection between concentration and velocity, is given in Eq. 5.

5 $$k{\mathrm{app}}*C=\frac{Kr*Ks*C}{1+Ks*C}$$

where *Ks* (L/mg) is the adsorption constant; *Kr* (mg/L.min) represents the rate of reaction.

Linear form of the equation:

6 $$\frac{1}{k{\mathrm{app}}}=\frac{1}{Kr*Ks}+\frac{Cr}{Kr}$$

1/*K*_aap_ is expressed as a function of Cr. Looking at the graph in Fig. 10, it is seen that the curve is linear (*r* = 0.9969). This shows that it is compatible with the L–H model.

### Time-dependent flow performance

The flow value of the PSCM mixtures produced within the scope of the study, measured every 20 min for 60 min, and the HRWRA requirement where the target flow value is achieved are shown in Table 5. Regardless of the utilization ratio and particle size, it was understood that the need for HRWRA increased in order to achieve the desired flow value with NT substitution into the mixture. In a similar study (Joshaghani et al. 2020), it was emphasized that the workability was adversely affected by the use of 5% NT, and this was due to the increased water requirement of the mixtures. Similar results were obtained by Nazari and Riahi (2010) and Nazari et al. (2010). According to Lee and Kurtis (2010), the use of NT in cementitious systems increases the hydration rate of C_3_S, causing the formation of C–S–H gel at earlier ages. It was emphasized that this situation causes an increase in the degree of hydration of cementitious systems and negatively affects the workability. Similar statements were also reported by Singh et al. (2015). In another study by Chen et al. (2012), it was found that when 5% and 10% NT are substituted into the mixture, the water requirement to achieve target flow value increases by 7 and 15%, respectively. It was emphasized that this may be due to the rapid free water consumption accelerating the bridging process of the voids, resulting in increased viscosity and earlier solidification. It was reported by Ma et al. (2016) that the addition of NT accelerates the formation and precipitation of early hydration products. It was emphasized that this situation caused the workability properties of NT-containing mixtures to be adversely affected.

**Table 5:** Time-dependent flow values of mixtures and HRWRA requirement

Mixture	HRWRA requirement (kg/m^3^)	Time-dependent flow values (mm)				Consistency retention capacity (%)
		0 min	20 min	40 min	60 min	
Control	3.5	220	170	160	150	68
NT28_0.5%	4.0	220	185	175	160	73
NT28_1.0%	4.3	235	165	160	155	66
NT28_1.5%	4.5	230	160	155	150	65
NT38_0.5%	3.5	240	170	160	155	65
NT38_1.0%	3.8	250	175	170	155	62
NT38_1.5%	4.0	245	170	160	155	63

It is also understood from Table 5 that the decrease in flow performance as a result of NT substitution into the mixture becomes more evident with the decrease in NT particle size. As a result of the addition of 1.5% of NT with 28- and 38-nm particle sizes to the mixture, it was understood that the need for HRWRA in PSCM mixtures increased by 29% and 14%, respectively. It is thought that the need for HRWRA is higher due to the increase in the wettable surface area (Durgun et al. 2022; Mardani-Aghabaglou et al. 2018; Özen et al. 2021; Şahin et al. 2022, 2020; Şahin and Mardani 2023b) and the amount of adsorbed water due to the increased surface area due to the decrease in NT particle size (Kuo et al. 2006). It was reported by Chen et al. (2012) that the rapid hardening potential, which is directly dependent on the NT particle size and utilization ratio, directly affects the consistency retention capacity of the mixtures.

It is understood from the table that 0.5% substituted PSCM mixtures in both particle sizes exhibited the best consistency retention performance at 60 min. In addition, it was observed that mixtures containing 0.5% NT have better consistency retention performance, regardless of particle size. It was observed that the consistency retention capacity decreased as the NT substitution ratio increased. It was reported by Wang et al. (2018) that this may be due to the increase in the hydration rate and the acceleration of the setting due to the increase in NT substitution.

### Mechanical properties

#### Compressive strength

**Fig. 11:**
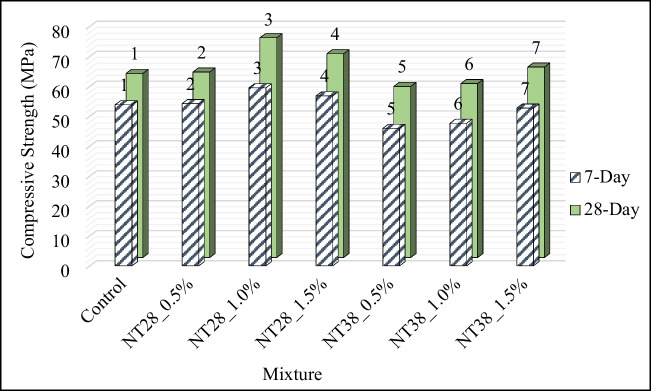
Seven- and 28-day CS values of PSCM

Compressive strength (CS) results of PSCM are shown in Fig. 11. It was understood that the CS of the mixtures containing NT with a lower particle size value (28 nm) was higher regardless of the sample age. This condition is believed to be the result of the quick consumption of Ca(OH)_2_ produced during the hydration of portland cement, which was brought on by the NT’s smaller particle size, increased surface area, and increased reactivity. However, it was stated by Nazari et al. (2010) that the use of NT improves the particle packing density of the cement and thus less large pores are formed in the cement paste.

It was observed that the 7-day CS increased by 1, 11, and 6%, respectively, with 0.5%, 1.0, and 1.5% NT substitution with 28-nm particle size to the control mixture. In 28-day samples, this rate was found to be 1, 19, and 11, respectively. Thus, it was determined that the optimum NT particle size and utilization ratio in terms of CS were 28 nm and 1%, respectively. It was understood that the use of NT above this ratio affects the CS of PSCM mixtures negatively. It was emphasized that this situation may be due to the decrease in the crystalline Ca(OH)_2_ content required for C-S–H gel formation with the increase of NT, on the one hand, and the increase in the amount of voids in the matrix as a result of the presence of large amounts of nano-particles in the system (Sorathiya et al. 2017). Similarly, in a study where NT with a particle size of 15 nm was used at 0.5, 0.75, 1, 1.25, and 1.50% of the cement weight, it was emphasized that the optimum NT usage rate in terms of CS was 1% (Sorathiya et al. 2017). In another study by Nazari et al. (2010), it was determined that the CS value obtained when 2% of NT with a particle size of 15 nm is used is similar to the control mixture without NT. According to reports, the combination contains more NT particles than is necessary for them to react with the lime that is produced during the hydration process. This results in excessive particle leakage and negatively affects the mixture’s strength. Similar results were also reported by Dikkar et al. (2021). In another study conducted by Mohd Sani et al. (2022), it was reported that the CS increased with an increase of 1% in the use of NT, but decreased above this rate. However, in a study conducted by Sharma et al. (2019), it was observed that the maximum CS value in mixtures containing NT with a particle size of 15 nm is obtained when 1.5% NT is used.

Various studies in the literature (Feng et al. 2013; Khataee et al. 2013; Karapati et al. 2014; Zhu et al. 2022; Mousavi et al. 2021) have emphasized that the optimum NT usage rate in cementitious mixtures containing NT is between 1 and 2%. In a study conducted by Zhu et al. (2022), the compressive strength of cement paste produced using different proportions of NT was examined. It has been reported that the mixture with optimum compressive strength contains 2% NT. In a study conducted by Khushwaha et al. (2015), the compressive strength of mixtures with 1%, 2%, and 3% NT content was examined. It has been determined that the strength decreases with increasing dosage. According to Chen et al. (2012), the compressive strength of mixtures containing two different NTs with particle sizes of 21 nm and 350 nm was examined. It was stated that the 7- and 28-day compressive strengths of the mixture containing 21 nm NT were higher than the results of the mixture containing 350 nm NT. It has been emphasized that the increase in strength is greater as the particle size decreases. However, the study conducted by Li (2021) reported the opposite. It was emphasized that as the particle size increases, it can absorb less amount of water as its surface area decreases. It was emphasized that NTs used in smaller particle sizes will deteriorate the W/B ratio, negatively affect workability, and cause internal defects. It was stated in various studies that the dosage of NT usage is important to increase the compressive strength in cementitious systems and that it depends on many parameters such as particle size, distribution, W/B ratio, and curing temperature (Li et al. 2023; Moro et al. 2020; Francioso et al. 2019, Pimenta Teixeira et al. 2016).

Unlike the mixtures containing NT with a particle size of 28 nm, the 7-day CS was decreased by 15, 12, and 2%, respectively, with 0.5%, 1.0, and 1.5% substitution of NT with 38-nm particle size in the control mixture. It was understood that this decrease was 7 and 6%, respectively, in the samples with 0.5% and 1% NT replacement for 28 days. A 3.5% increase in strength was measured for the mixture containing 1.5% NT. It is thought that this negative effect experienced with the use of NT with 38-nm particle size is due to its relatively lower surface area and reactivity compared to 28 nm NT.

#### Three-point flexural strength

**Fig. 12:**
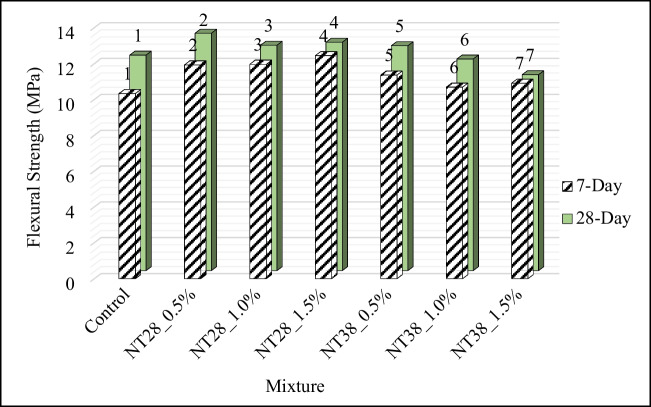
Results of FS of mixtures

Seven- and 28-day three-point flexural strength (FS) results of PSCM are presented in Fig. 12. FS values increased with the addition of NT to the mixture, regardless of particle size and sample age. It was understood that there are different opinions about this behavior in the literature. Nazari and Riahi (2010) and Nazari et al. (2010) suggested that this is due to the acceleration of the hydration reaction, especially at early ages, with the addition of NT, and thus the formation of hydration products in larger volumes. However, according to Meng et al. (2012), this increase in FS was not due to the increase in the amount of hydration products, which was due to the decrease in the orientation index for the core function. On the other hand, it was stated by Nazari and Riahi (2010) and Nazari et al. (2010) that NTs provide the reduction of voids in cementitious systems by recovering the particle packing density of the cement, thus contributing to the increase in strength. It was determined that the 7-day FS were increased by 15, 16, and 20%, respectively, with 0.5%, 1.0, and 1.5% NT substitution with 28-nm particle size to the control mixture.

In a similar study (Selvasofia et al. 2022), in which NT was used at 1, 2, 3, and 4% of cement weight, it was found that 2% and 3% NT substituted mixtures had the highest FS values. In order to explain the reason for this situation, FESEM images were examined. It was observed that in the 2% and 3% NT substituted state, a homogeneous C-S–H gel with relatively few spaces between the particles was formed. In addition, it was reported that NT used at the rate of 2% and 3% is fully bonded to the cement, resulting in a stronger bond and increased FS. However, it was understood that the highest FS values in the 28-day samples were obtained when the NT usage rate was 0.5% and it was not necessary to increase the usage rate. A similar statement was also stated by Meng et al. (2012).

In NT-substituted mixtures with 38-nm particle size, 4% and 13% reductions were measured in 7- and 28-day FS, respectively, with an increase in NT utilization rate from 0.5 to 1.5%. In a study conducted by Salman et al. (2016), the effect of using 0.25%, 0.75, 1.25, and 1.75% NT on the FS of mixtures was investigated. As a result, it was reported that the use of NT up to 0.75% causes the FS of the mixtures to increase, but the use of NT above this ratio causes the FS values to be adversely affected. It was stated that in the case of 0.75% of the cement weight NT is used, it acts as a filler to strengthen the microstructure of the system, reduces the amount and size of CH crystals, and increases the strength due to filling the voids of the C-S–H gel structure. However, it was reported that there is a decrease in nano-particle distance with an increase in the amount of NT use up to 1.75%, and the CH crystal cannot grow to the appropriate size due to the limited space, and this causes a decrease in strength.

As with the CS values, it was measured that the NT-containing mixtures with a particle size of 28 nm have a higher FS value than the mixtures containing NT with a particle size of 38 nm, regardless of the NT utilization rate. With smaller particle size, the number of surface atoms increases, depending on the increase in the surface area. It was emphasized that the highly active and unstable nature of these surface atoms causes an increase in the hydration reaction rate and higher strengths (Kuo et al. 2006).

In addition, it was observed that the optimum NT utilization rate was 0.5% in terms of FS, regardless of particle size. In this case, the mixture with the highest FS was NT28_0.5%.

#### Bohme abrasion resistance

**Fig. 13:**
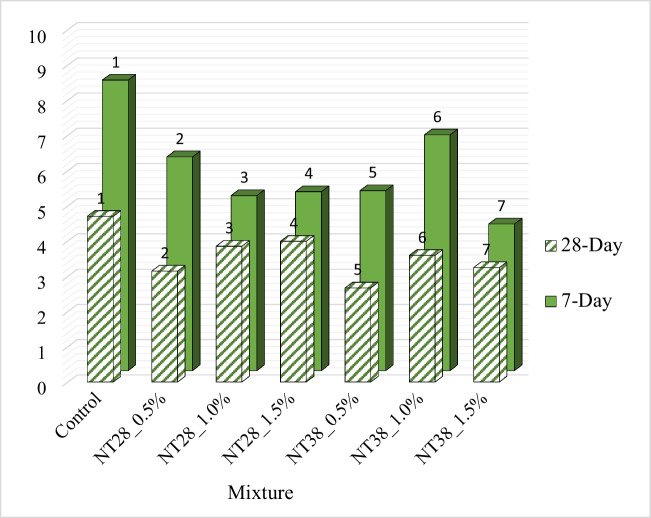
Bohme abrasion resistance results

The 7- and 28-day Bohme abrasion resistance (B’AR) results of PSCM specimens are shown in Fig. 13. It was found that mixtures containing NT had higher B’AR at all ages, independent of NT particle size and utilization ratio. This situation was thought to be due to the formation of a denser matrix with the use of nano-materials and the reduction of the permeability of the system due to the nucleation effect (Selvasofia et al. 2022). A study by Chen et al. (2012) reported that as hydration continues, aggregations containing nano-particles expand and fill the void space around them over time, contributing to the reduction of porosity over time. On the other hand, Gartner et al. (2002) emphasized that the decrease in porosity as a result of nano-material addition is due to the physical clogging of capillary spaces.

Effect of NT usage rate on the B’AR of the mixtures varies depending on the sample age, particle size, and NT usage rate. It was observed that the mass loss of 7-day PSCM decreased with the increase in the use of NT with 28-nm particle size up to 1%. However, the opposite was found with NT substitution above this ratio. It was understood that the mass loss increased continuously with the increase in the use of NT in the 28-day samples. In mixtures containing NT with a particle size of 38 nm, regardless of the sample age, it was understood that the mass loss increased as the NT usage rate increased up to 1%, but decreased above this rate.

It was found that NT substituted mixtures with 38-nm particle size have higher B’AR than mixtures containing 28 nm NT. This is thought to be due to the increase in the particle size of NT and the NTs that do not bind to CH leak onto the surface (Nazari and Riahi 2010; Nazari et al. 2010), contributing to the hardening of the surface and thus to the increase in B’AR. When the 7-day B’AR performance was examined, it was observed that the NT38_1.5% mixture had a 50% higher B’AR compared to the control mixture. This result was measured to be followed by the NT28_1.0% mixture, which had a 40% increase compared to the control. In the 28-day samples, the mixture with the best B’AR was found to be NT38_0.5% (44% increase compared to control), followed by NT28_0.5% (33% increase compared to control).

## Microstructure ımages

**Fig. 14:**
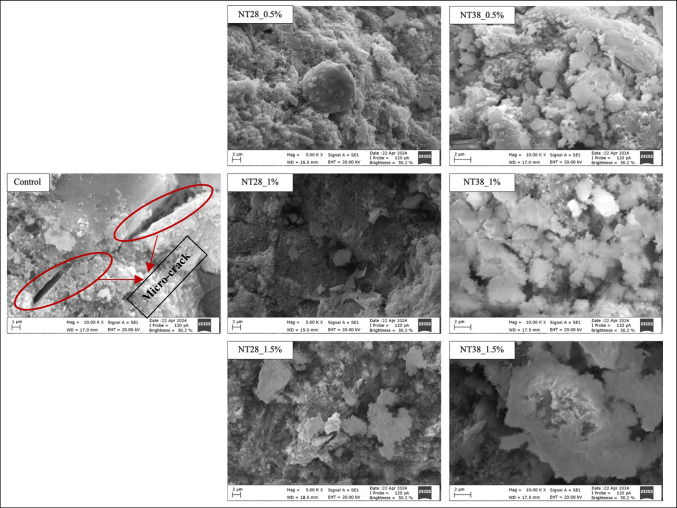
Microstructure images of mixtures

Microstructure images of the mixtures produced within the scope of the study are shown in Fig. 14. The micro-structure of the specimens was studied by using Carl Zeiss/Gemini 300 electron microscope. Scanning electron microscopy (SEM) analyses were attempted to identify the properties of mentioned materials. When Fig. 14 is examined, the effect of NT addition on density and porosity is clearly understood. It was observed that the cracks and pores in the control mixture, shown with the red line, decreased with the use of NT, regardless of the fineness of the NT. Similarly, in a study conducted by Feng et al. (2013), cement paste containing 0.1, 0.5, 1.0, and 1.5% of the cement mass with NT and a water/cement ratio of 0.4 was prepared and SEM images were examined. As a result, it was observed that NT substitution greatly reduced the amount of internal microcracks in the cement paste. Also, it was emphasized that a new type of needle-shaped hydration product was observed.

## Conclusions

The following results are provided in line with the study’s materials and the practical experiments:

- It was understood that the mixtures containing NT with a higher particle size value (38 nm) in terms of photocatalytic properties performed better.
- It was determined that decolorization kinetics were compatible with Langmuir–Hinshelwood model in all mixtures regardless of NT particle size and utilization ratio.
- It was understood that the flow performance of PSCM mixtures was negatively affected by NT substitution. This situation became more evident with the increase in NT utilization ratio and the decrease in particle size.
- It was determined that the addition of 1.5% of NT with particle sizes of 28 and 38 nm to the mixture raised the requirement for HRWRA in PSCM mixes by 29% and 14%, respectively.
- CS of the mixtures containing NT with lower particle size (28 nm) was higher. In terms of compressive strength, the optimum NT particle size and utilization ratio were found to be 28 nm and 1%, respectively.
- It was found that when 0.5%, 1.0, and 1.5% of the NT replacement with 28-nm particle size was added to the control mixture, the 7-day CS increased by 1, 11, and 6%, respectively. This rate was determined to be 1, 19, and 11 in 28-day samples.
- It was determined that flexural strength and Bohme abrasion resistance values increased with the addition of NT to the control mixture, regardless of particle size and sample age. In terms of flexural strength, the optimum NT utilization rate was 0.5%. NT-substituted mixtures with 38-nm particle size were measured to have higher abrasion resistance.
- It was found that when 0.5%, 1.0, and 1.5% of the NT replacement with 28-nm particle size was added to the control mixture, the 7-day CS increased by 1, 11, and 6%, respectively. This rate was determined to be 1, 19, and 11 in 28-day samples.
- When the 7-day B’AR performance was examined, it was measured that the NT38_1.5% mixture had a 50% higher B’AR value than the control mixture, and the NT28_1.0% mixture showed a 40% increase compared to the control.

## Data Availability

The data that support the findings of this study are available on request from the corresponding author.
